# Continuing chronic care services during a pandemic: results of a mixed-method study

**DOI:** 10.1186/s12913-022-08380-w

**Published:** 2022-08-08

**Authors:** Jennifer Sumner, Anjali Bundele, Lin Siew Chong, Gim Gee Teng, Yanika Kowitlawakul, Amartya Mukhopadhyay

**Affiliations:** 1 Yong Loo Lin School of Medicine, National University of Singapore, Singapore, Singapore; 2 Medical Affairs – Research Innovation and Enterprise, Alexandra Hospital, National University Health System, Singapore, Singapore; 3 Department of Health Sciences, University of York, York, UK; 4 Chronic Programme, Alexandra Hospital, National University Health System, Singapore, Singapore; 5 Department of Medicine, Division of Rheumatology, National University Health System, Singapore, Singapore; 6 Alice Lee Centre for Nursing Studies, Yong Loo Lin School of Medicine, National University of Singapore, Singapore, Singapore; 7 Division of Respiratory and Critical Care Medicine, Department of Medicine, National University Hospital, Singapore, Singapore

**Keywords:** Chronic disease management, Ambulatory care, COVID-19, Self-management, Health services research

## Abstract

**Background:**

Patients with chronic diseases have seen unprecedented changes to healthcare practices since the emergence of COVID-19. Traditional ‘on-site’ clinics have had to innovate to continue services. Whether these changes are acceptable to patients and are effective for care continuation are largely unreported.

**Methods:**

We evaluated the effectiveness of care provision at a re-structured chronic care clinic and elicited the patient experiences of care and self-management. We conducted a convergent, parallel, mixed-methods study. Adult patients attending a chronic care clinic were included. We extracted data from 4,849 clinic visits before and during the COVID-19 pandemic, including operational metrics and attendee profile. We also conducted fifteen interviews with patients from the same clinic using a semi-structured interview guide.

**Results:**

Re-structuring the chronic clinic, including the introduction of teleconsultations, home-delivery of prescriptions and use of community-based phlebotomy services, served to maintain continuity of care while adhering to COVID-19 containment measures. Qualitatively, five themes emerged. Patients were able to adjust to healthcare practice changes and adapt their own lifestyles, although poor self-management practices were adopted. While most were apprehensive about attending the clinic, they valued ongoing care access and were reassured by the on-site containment measures.

**Conclusions:**

Continuation of routine services is desired by patients and can be achieved through the adoption of containment measures, by greater collaboration with community partners, and the use of technology. Patients adapted to service changes, but poor self-management was evident. To prevent chronic disease relapse, services must strive to innovate rather than suspend services during pandemics.

**Supplementary Information:**

The online version contains supplementary material available at 10.1186/s12913-022-08380-w.

## Background

COVID-19 has caused widespread disruption to healthcare services around the world [1, 2], following the rapid spike in cases [3] and the frequent requirement for hospitalisation [4]. To prevent healthcare providers becoming overwhelmed by COVID-19 cases, many countries introduced strict containment measures. Country borders were closed, remote working became commonplace, and social distancing practices were implemented [5, 6]. In Singapore, a country wide ‘lock-down’ (locally known as the ‘circuit-breaker’) was introduced in April 2020 [6]. Residents were advised to remain indoors except for essential trips, wear masks while outside, work or school from home, and have no interaction with those in other households [6]. Concurrently, healthcare institutions increased their capacity for COVID-19 management through the reallocation of staff and facilities, suspending non-urgent appointments and procedures, and the introduction of teleconsultations [1, 2, 7–10].

While the reorganisation of healthcare services helped to increase the capacity to manage COVID-19 patients in the short-term, it has disproportionately impacted those requiring less urgent long-term care (i.e., patients with chronic disease). Patients with chronic disease(s) are the highest users of healthcare services [11]. Proper healthcare access, coordination, and continuity of care are essential to effective chronic disease management [12]. Without sustained care and good self-management, poorly controlled chronic disease may arise and lead to emergency department visitations and hospital admissions [13, 14]. Thus, maintaining access to care is a priority.

At Alexandra Hospital, an outpatient clinic attempted to maintain safe access to chronic care (during the pandemic) by implementing several containment strategies. Patients visiting the clinic had to undergo symptom screening prior to clinic entry, accompanying carers were limited to one, and the seating arrangements in the waiting area were redesigned to maintain social distancing [15, 16]. Teleconsultations and home-delivery of prescriptions were introduced in lieu of face-to-face appointments and patients were given the option of having blood tests in community-based clinics rather than at the hospital [15, 16]. Although containment measures have worked to protect patients from infection, the wider implications of practice changes are not entirely known.

To date, studies have largely focused on the provider perspective when evaluating modified chronic care services, rather than the patient experience [17–19]. There is a need to properly assess the impact of COVID-19 on patient care and lifestyle, to ascertain whether patient needs are still met, effective care is delivered, and if patients can self-manage their chronic conditions.

The purpose of this study was to investigate if effective care provision was sustained following the restructuring of chronic care services to accommodate COVID-19 containment measures. We also sought to explore the lived experiences of those attending the redesigned clinic. Our aims were:

1. To investigate if chronic care services were maintained following clinic restructuring (due to COVID-19).
2. To explore the patients’ experiences of the restructured chronic care service and their own self-management, since COVID-19 emerged.

## Methods

A convergent, parallel, mixed-methods study design was conducted. The mixed-method design was chosen so quantitative and qualitative data could be collected and triangulated to improve the validity of the findings. Qualitative data were collected and reported according to the COREQ checklist (Consolidated criteria for reporting qualitative research) [20].

The study was approved by the National Healthcare Group Domain Specific Review Board (NHG DSRB: 2020/00303).

### Quantitative data collection

Data were extracted from the electronic medical records of the outpatient chronic care clinic at Alexandra Hospital between 10^th^ November 2019 to 7^th^ May 2020. The data were then analysed separately for a 3-month period before COVID-19 measures were implemented (10^th^ November 2019-7^th^ February 2020) and for a 3-month period after clinic restructuring due to COVID-19 (8^th^ February 2020-7^th^ May 2020). Data included operational metrics from the outpatient chronic care clinic (number of appointments, type of visit, number of teleconsultations, grade of treating physician, referral source, number of radiology orders, and number of laboratory orders) and the demographics of patients attending the clinic during the study period.

### Statistical analyses

Analyses were performed in STATA v15.0 (STATA Corp, College Station, Texas, USA). Summary statistics are presented as mean (with standard deviations, SD) or proportions. The patient profile was compared before and after clinic restructuring, using a two-sample t-test or Chi^2^ test as appropriate.

### Qualitative data collection

Qualitative data was collected through fifteen semi-structured interviews with patients between 24^th^ April- 2^nd^ June 2020. Patients were recruited from the restructured outpatient chronic care clinic at Alexandra Hospital. Eligible participants were adults (≥ 21 years) with at least one chronic disease. Patients with different chronic conditions were recruited to ensure a breath of views (i.e., purposive sampling). Participants with cognitive impairment were excluded.

Initially, eligible participants were identified by doctors running the chronic care clinic. Participants willing to participate were consented and their contact details were conveyed to the interviewers. Two female researchers, AB (BDS, MPH) and LSC (BSc, MSc), conducted the interviews in English, Chinese, or Malay, as per the participant’s preference. Both researchers were trained in qualitative research methodologies and were proficient in the language spoken by the participants. The interviewers had no direct or dependent relationship (patient-doctor) with the participants, which could potentially influence responses. Due to COVID-19 containment measures, interviews were conducted remotely via telephone or videoconference (Zoom). Remote interviewing is a suitable alternative when face-to-face interviewing is not practical [21, 22]. The interview was conducted between the interviewer and the patient alone or alongside the caregiver. Before the interview commenced, the study aim was reiterated and permission was sought for audio-recording and transcription of the discussion. A total of twenty-one participants were invited for interview, and fifteen interviews were conducted and analysed.

A semi-structured interview guide was initially developed with reference to the chronic care model [12]. The chronic care model describes six components for effective chronic care management. An initial set of questions was discussed and then refined with clinicians who manage chronic disease patients (Supplementary file 1). The interview guide contained a series of open-ended questions, with prompts where necessary. Interviews took between 30 to 50-min. After each interview, the interviewers reflected and generated memos to aid with analyses.

### Qualitative analyses

All audio recordings were transcribed and translated into English. The accuracy of the translation was checked by a second independent researcher. Data were analysed using a thematic analysis method, which includes coding the data and then developing sub-themes and main themes [23]. Data were coded according to the meaning of the sentences to identify experiences as perceived by patients. Similar and overlapping sub-themes were then grouped under main themes. Coding was conducted in MS Office by JS, AB and LSC independently. The interpretation of each transcript was then discussed as a group and differences in opinion were mutually reconciled. Interviews and coding occurred concurrently until data saturation was reached.

### Quantitative and qualitative synthesis

Data were triangulated by first analysing the quantitative and qualitative results separately. Through discussion, members of the research team (JS, AB, LSC) then compared the key points from the quantitative data to the sub-themes and themes of the qualitative data. Areas of commonality between the quantitative and qualitative results were identified and are summarised in the discussion. The approach allowed us to bring a greater depth of meaning to the quantitative findings.

## Results

During the study period, data from *n* = 4,849 clinic visits (2,500 visits before clinic restructuring due to COVID-19 and 2,349 visits after restructuring) were analysed and fifteen qualitative interviews were conducted.

### Quantitative results

The number of appointments remained relatively stable. The patient profile did not statistically significantly change before and during the COVID-19 period (Table 1). Presenting diagnoses remained similar. Of the top five diagnoses before and after COVID-19 (based on the International Classification of Diseases (ICD)-10 [24]), the first four remained the same: (E00-E99) endocrine-related, (I00-I99) circulatory-related, (M00-M99) musculoskeletal-related, (R00-R99) general signs, symptoms and abnormal findings. The fifth most common diagnosis changed from (K00-K95) digestive-related to (N00-N99) genitourinary-related.

**Table 1:** Patient demographics and clinic operational data before and during the COVID-19 pandemic period

	**Before COVID-19** **10**^**th**^** Nov 2019– 7**^**th**^** Feb 2020** **(*n* = 2,500 clinic visits)**	**During COVID-19** **8**^**th**^** Feb-7**^**th**^** May 2020** **(*n* = 2,349 clinic visits)**	***p* value**
Mean age, years (range, SD)	61.53 (18–101,18.28)	60.76 (14–101,18.23)	0.14
Female, n (%)	1,348 (54)	1,222 (52)	0.18
Ethnicity, n (%)			
Chinese	1,856 (74)	1,736 (74)	0.39
Indian	241 (10)	213 (9)	
Malay	211 (8)	229 (10)	
Other	192 (8)	171 (7)	
Marital status, n (%)			
Married	1,007 (40)	998 (42)	0.48
Single	315 (13)	279 (12)	
Other (unknown etc.)	1,178 (47)	1,072 (46)	
First visit, n (%)	739 (30)	687 (30)^a^	0.65
Return visit, n (%)	1,761 (70)	1,592 (70)	
Grade of doctor, n (%)			
Senior consultant	412 (16)	356 (15)	< 0.001
Consultant	1,192 (48)	1,317 (56)	
Associate consultant	805 (32)	578 (25)	
Non-doctor consultation	91(4)	98 (4)	
Referral from, n (%)			
Polyclinics or community clinics	977 (39)	807 (35)	< 0.001
Within Alexandra hospital	1266 (51)	1080 (46)	
Other hospitals	211 (8)	431 (18)	
Other clinics/private care	46 (2)	31 (1)	
Number of radiology orders, n	4,253	5,170	
Number of laboratory orders, n	14, 431	16,713	

*Abbreviation*: *SD* Standard deviation, ^a^missing data *n* = 70

Statistically significant changes in the grade of staff treating patients and the referring location were observed. Patients were more likely to be treated by consultant grade staff rather than associate consultants. The number of referrals from other hospitals also increased during the COVID-19 period. In terms of diagnostics, both radiology and laboratory orders increased during COVID-19. Finally, teleconsultation appointments were introduced in February 2020. A total of five appointments occurred in February, increasing to ninety appointments by May 2020.

### Qualitative results

Table 2 presents the demographical profile of the fifteen participants interviewed. Over two-thirds of participants had two or more chronic conditions. Ten participants were referred to the outpatient chronic care clinic from the hospital setting; the remaining came from community referrals (i.e., General Practitioners).

**Table 2:** Qualitative participant profile

	***n* = 15**
Mean age, years (range, SD)	61.20 (38–80, 12.90)
Female, n (%)	9 (60)
Ethnicity, n (%)	
Chinese	11 (73)
Indian	3 (20)
Malay	1 (7)
Highest education level, n (%)	
No formal/primary level education	1 (7)
Secondary	7 (47)
A Level	2 (13)
Diploma and above	5 (33)
Marital status, n (%)	
Married	11 (73)
Single	3 (20)
Divorced	1 (7)
Employment status, n (%)	
Full-time	9 (60)
Homemaker	1 (7)
Retired or unemployed	5 (33)
Medical history, n (%)	
Rheumatoid arthritis	5 (33)
Gout	4 (27)
Systemic lupus erythematosus	3 (20)
Diabetes mellitus	3 (20)
Osteoporosis	3 (20)
Hypertension	2 (13)
High cholesterol	2 (13)
Heart disease	2 (13)
Asthma	1 (7)

*Abbreviations*: *SD* Standard deviation

Five main themes emerged from the analysis of the interview data.

#### Theme 1: adapting lifestyle in the COVID-19 era

During the interviews, most participants reflected on several changes to their lifestyle because of COVID-19. Adaptations included an increase and preference for more home cooking, an avoidance of grocery shopping, and adoption of home-based exercise.

*ID16: “Normally, before COVID at least I can do some exercise outside but now not outside, I just do simple exercise at home”*

Participants reported feeling nervous about catching the disease, particularly if they had an underlying condition. Concerns around getting COVID-10 appeared to be the main driver of personnel lifestyle adaptations. Some lifestyle adaptations were also imposed on participants due to the containment measures (i.e., closure of communal spaces, no in-restaurant dining, no socialising between households, and working from home). In some cases, participants felt pressure from relatives to limit their interactions outside the home.

*ID5: “At the beginning of the outbreak, I felt nervous. I have heart disease, SLE, have problems in [my] immune system…stay at home, avoid going out and get infected from people out there”*

*ID03: “I cannot meet family members now since they are staying at different places. There has been no physical meeting since then [since COVID-19]. Only through phone, there is no face-to-face interaction”* [households were not permitted to mix during lock-down]

*ID15: “The young one will say don’t go out if you need anything…but sometimes they buy the thing, it’s not what we want”*

*ID7: “My son also doesn’t want us go to out, so he will order online”*

#### Theme 2: finding reassurance from COVID-19 containment measures

Most participants were concerned about the pandemic and were apprehensive about visiting the clinic. Anxiety about visiting the clinic eventually resolved after seeing the doctor in the hospital and experiencing the containment measures. For most, containment measures were felt to be sufficient, and they understood their requirement.

*ID8: Of course, they are necessary. To protect yourself, your family, and others. You never know when the person next to you may…show no symptoms”*

*ID7: “At the beginning, really worried. After first and second visits, I felt like…knowing that they are doing precaution measures, then won’t felt so scared”*

While participants were knowledgeable about COVID-19 and the associated containment measures, the clinic was not viewed as a source of information or advice regarding COVID-19.

*ID2: “They did not specifically explain, but we will understand by ourselves”*

[in relation to COVID-19 information provision by the clinic]

*ID11: “I mean, it will be good if we have more information with regards to what precautions other than the very general precautions that we should take”*

#### Theme 3: accessibility of Healthcare despite COVID-19

Participants generally described positive experiences with healthcare, reflecting continued access and continuity of care in the clinic. Most participants stated that their appointment frequency was unchanged, although many participants' appointments were temporarily moved to another institution (physicians were prohibited from practicing at multiple institutions during lock-down). While continued access to care was viewed favourably and participants were generally satisfied, there was some frustration at the inconvenience of changing location.

*ID7: “Still can go see a doctor.”*

*ID11: “err, of course it is inconvenient, but I guess if it really is for some good reason then I’m fine with it”[changing clinic location]*

Due to COVID-19, many healthcare institutions utilised teleconsultations in place of face-to-face appointments. Generally, this was an acceptable substitute for onsite consultations.

*ID12: “yeah, I think it's a good idea, then we save travelling, and it's safer also”*

In other cases, a lack of technological ‘savviness’, the absence of ‘personal touch’, and scepticism regarding its effectiveness were reported as barriers to adoption. Personnel context also appeared to influence the acceptability of teleconsultation (e.g., current disease status).

*ID15: “I rather go there and wait for the doctor to see me…for this skill [using teleconference], it’s quite difficult, all the time I got to get someone to help me”*

*ID7: “If my condition allows, then I will accept. If my condition gets worse, then I cannot”*

*ID14: “I think there won't be any personal interaction, and it would be like you're talking to the machine even though the doctor is zooming you (Laughs). I prefer to talk face to face…because you can see the reaction of the person”*

#### Theme 4: anxiety due to COVID-19

Participants reported feelings of anxiety for themselves and others. They expressed concerns for the future, the economic impacts of COVID-19, and healthcare worker shortages.

*ID16: “I hope everything will be fine, pity for other people, for children that cannot go out and gather with friends”*

*ID17: “Due to the economy so bad, I don't want to see it continue…because let's say if I continue, so many months or half a year to work from home, it really affects is very challenging, umm in terms of I don't know whether my work, it can keep on”*

*ID7: “If our doctors get infected, the hospital will be lacking doctors, nurses or healthcare workers… we do not want to spread our disease to them.”*

Feelings of negativity and frustration with the current situation were common. There was also a strong desire for things to return to normal post COVID-19.

*ID13: “I also hope that this disease faster goes away, everyone can go back to a normal life. I hope everything will be fine after this, no more lock-down, hopefully things will be turn back to normal”*

#### Theme 5: Resilience in lock-down

A strong sense of resilience surfaced in the interviews. Participants adapted to COVID-19 related changes, using various coping strategies. Many adopted technological solutions (e.g., telecommunications) so they could continue to socialise while avoiding activities perceived as high risk.

*ID15: “Because of COVID-19 we are not supposed to meet in church, then no choice lah…sometime attend on YouTube lah, the sermon on Youtube”*

*ID14: “I have two good friends, and we meet once every month. But now that has been banned, so we contact through phone”*

(“lah” is a commonly used phrase in local English dialect (often termed Singlish) which may mean an affirmation, dismissal, or exclamation in different contexts.)

Trust in the government’s actions, and an understanding that ‘the restrictions are for our own good’ also helped participants accept the situation and remain resilient.

*ID7: “Our Singapore did very well in terms of precaution measures, my feelings become more calm. At the beginning, will feel nervous. But now the government will control it, so we won’t feel so worried now”*

## Discussion

We investigated the continuity of chronic care services (following clinic re-structuring) and explored the lived experiences of those attending the redesigned clinic. We used a mixed-method approach, combining clinic operational data with qualitative interviews. We found that restructuring the clinic (to enhance safety) had little impact on the clinic’s operationing metrics. No substantial changes in the number of appointments, type of attendee (age, sex, ethnicity, marital status, diagnoses) or type of hospital visit (first or follow-up) were observed. Interviewed patients expressed satisfaction in the continued access to routine healthcare services. Participants valued the ability to proceed with appointments as per normal (despite COVID-19) and adapted to changes in the clinic set-up, as well as safe distance practices in the community.

Before attending the clinic, many participants reported a sense of apprehension as to what to expect; ultimately this did not deter patients from their appointment (as demonstrated by similar appointment numbers during the two periods). Rather, participants eventually felt reassured by the safety measures in place at the clinic. The fact that patients were well informed about COVID-19 and understood the need for the associated containment measures likely influenced clinic attendance positively. While we found patients understood how and why COVID-19 is being managed, they did not view the hospital clinic as a source for COVID-19 related information. This fits with a recent survey (in Singapore), which reported social media, television programmes and friends or colleagues are the main sources of COVID-19 related information [25]. We cannot comment on the accuracy of the COVID-19 information that patients obtained in this study, but widespread reports of misinformation (particularly through social media) should be acknowledged [26]. Clinic visits may be an ideal opportunity for healthcare providers to play a larger role in providing reliable and accurate information to patients, although a few barriers remain. Identifying who would benefit from information provision and how to tailor the information to individuals takes time. Additionally, COVID-19 related information is continually evolving, while clinic visits are spaced in time, providing timely communications through the clinic may therefore be problematic.

For patients not physically attending the clinic, video teleconsultation became an option as part of clinic restructuring. Patients recognised the need and benefits of this approach, reporting on the convenience of remote appointments and the ability to feel safe by staying away from the hospital. Conversely, some doubted the use of teleconsultations due to a perceived lack of skill or support at home, a belief that teleconsultation is impersonal or ineffective, and that teleconsultation is only ‘OK’ while COVID-19 persists. From the providers' perspective, adoption of teleconsultation is desirable in that it avoids physical visits (reducing the risk of infection) and minimises the use of scarce personal protective equipment [27]. In similar studies investigating healthcare disruptions due to COVID-19, digital solutions (i.e., teleconsultations) have been suggested as a way to reorganise healthcare and maintain care access [17, 18]. Indeed, global data reflects an accelerated adoption of teleconsultation by health systems [28]. However, care must be taken to avoid worsening care inequalities [19]. Well reported barriers to telemedicine use must be addressed for it to be effective [29]. Virtual care policies must also acknowledge that video teleconsultations are not appropriate for every patient or circumstance, a point reflected in our interviews.

Outside the healthcare setting, patients reflected on the many lifestyle adaptations they made for themselves or experienced in the community. Patients perceived that they were able to adjust well during the COVID-19 outbreak. However, it was apparent that many of the reported lifestyle adaptations were poor substitutes. For instance, when residents were advised to remain indoors as much as possible, many stopped exercising. If home-based exercise was performed the intensity was reduced. Other lifestyle changes included a loss of autonomy, with families not wanting their senior parents [interviewees] to leave home or shop for themselves. Participants also mentioned a reduction in food choice (when home-cooking) or having to use fewer fresh ingredients due to stockpiling. COVID-19 related anxiety also appeared to play a role in how participants adapted their lifestyle. Many reported that they did not need to worry if they stayed at home, but this came at the cost of not socialising or exercising. While it is encouraging that patients were able to adapt their lifestyles, the adoption of poorer habits is concerning. Effective disease control requires good self-management, as recognised in Wagner’s chronic care model [12]. Patients with chronic diseases must be supported by healthcare providers to continue healthy self-management practices, even during disease outbreaks.

While our study has many strengths, there are limitations. Firstly, the analysis was based on data from one institution. The operational practices may be different elsewhere and may not reflect the whole of Singapore. For instance, some hospitals suspended outpatient care during the peak of the pandemic, while the clinics at Alexandra Hospital were able to operate to some extent. Furthermore, we only recruited patients from the hospital outpatient clinics. While their experiences are valid, they may not reflect the wider populations' experiences. Secondly, our retrospective analysis was limited by what variables were captured and could be extracted. Some relevant operational metrics were not available, which may mean we do not have the complete picture of how well the outpatient clinics performed. For example, we were unable to look at appointment cancellations at the time of this analysis. Although we could not quantify appointment cancellations, we were able to explore patient preferences for ongoing care qualitatively. Finally, due to the duration of the study we are unable to comment on the long-term consequences of COVID-19 on healthcare practices and any lasting implications for patients with chronic conditions. Future studies should explore the longer-term implications of COVID-19 on healthcare and patient outcomes.

## Conclusion

COVID-19 has caused profound changes to the delivery of routine healthcare for chronic disease patients. Through careful adoption of containment measures, greater collaboration with community partners, and the use of technology, the continuation of routine outpatient services is feasible and desired by patients. Patients are adaptable to changes in clinic structure, and many of the service innovations have enhanced care beyond the pandemic. However, poor lifestyle practices were identified. This finding emphasises the important role healthcare providers must play in continued self-management support. To prevent disease relapse, healthcare must strive to innovate rather than suspend services during pandemics.

## Supplementary Information

**Additional file 1: Supplemental file 1.** Semi-structured interview guide.

## Data Availability

The data are available from the corresponding author upon reasonable request.

## Abbreviations

COREQ: Consolidated criteria for Reporting Qualitative research
ICD: International Classification of Diseases
SD: Standard Deviation
