# Supplementary material for: Continuing chronic care services during a pandemic: results of a mixed-method study
Source: BMC Health Serv Res. 2022 Aug 8;22:1009. doi: 10.1186/s12913-022-08380-w (PMC9358920; doi:10.1186/s12913-022-08380-w)
Supplement: Supplementary file 1 — Additional file 1: Supplemental file 1. Semi-structured interview guide. [file 12913_2022_8380_MOESM1_ESM.docx]

Supplemental file 1: Semi-structured interview guide

Continuing chronic care services during a pandemic:

Results of a mixed-method study.The Patient – Patient Centered Outcomes Research

Jennifer Sumner, [jenny.sumner@york.ac.uk](mailto:jenny.sumner@york.ac.uk)

**In-depth Interview Topic Guide**

**COVID-19 and the impact to chronic care**

1. Tell me about your experiences of the hospital clinic care since the COVID-19 outbreak started?

(Prompts: Number of appointments, rescheduling of appointment, change in duration/venue/doctor-in-charge of the appointment, obtaining prescription/medication)

1. How do you feel about it?
2. Do you think there have been impacts on your health since the COVID-19 outbreak started?

(Prompt: If yes, what are they)

1. How do you feel about the additional safety measures at the hospital premises?

(Prompts: health screening at registration, telephone screening day prior to visit, prolonged time spent in the hospital campus, perceived effectiveness/necessity of measures)

1. Are there any additional measures that you think could be implemented?
2. Tell me your views on the information provided by the hospital clinic on COVID-19?

(Prompts: enough and at the right level, patient specific)

1. Have you had a tele-consultation yet? If Yes, how has the experience been?

(Prompts: consultation duration adequate, ability to self-manage remotely, language barrier faced, technological barrier faced, cost of consult same as face to face)

1. Thinking about the current COVID-19 situation, has your usual lifestyle changed in any way?

(Prompts: physical activity, healthy eating, social activities)

1. What have been your experiences of self-managing (i.e., looking after your own condition, life-style practices) during this period?

(Prompts: feelings positive/negative, access to care, disease control, medication use/access, adequate resources and support available?)

1. Considering the COVID-19 situation, what (if any) are your additional needs in order to self-manage well?
2. Is there anything further you would like to raise or discuss?
